# A Genetic Basis for Hypersensitivity to “Sweaty” Odors in Humans

**DOI:** 10.1371/journal.pbio.0050298

**Published:** 2007-10-30

**Authors:** Liza Gross

Conventional wisdom and comparative genomics both suggest that the human sense of smell pales in comparison to the olfactory skills of our canine and rodent relatives. Humans and mice have hundreds olfactory receptor genes, but mutations in our olfactory genes have turned the vast majority into nonfunctional “pseudogene” relics. Nonetheless, humans can detect as many as 10,000 different odors, although individual olfactory sensitivities are highly variable. About 2 million people in the United States have no sense of smell (a disorder called general anosmia), though “specific anosmia”—insensitivity to a particular odor—is more common. One in ten people reportedly can't smell hydrogen cyanide (a poisonous gas), while 12% of participants in one study failed to detect musky odors, common perfume ingredients. An estimated one in 1,000 lucky souls can't smell butyl mercaptan, the rancid issue of skunks. At the opposite end of the spectrum are the hyperosmics, who can detect minute traces of odors such as androstenone (a steroidal component of human saliva, sweat, and urine).

General anosmia is rarely genetic and more commonly results from head injury, allergy, or nasal infection. Specific anosmia is thought to arise from mutations in olfactory receptor genes. Embedded in the sensory neurons of the nasal epithelium, olfactory receptors transmit information about a chemosensory molecule (an odor) to other neurons in the brain's olfactory bulb. Researchers have long suspected that variations in the genes that encode olfactory receptors, which function on the front lines of odor recognition, may explain the vast differences seen in humans' ability to detect odors. And now, in a new study, Idan Menashe et al. have identified an olfactory receptor gene that confers a high sensitivity to the “sweaty” odorant, isovaleric acid.

Menashe et al. explored the genetic basis of human olfactory diversity by focusing on a subset of olfactory receptor genes with widespread mutations (single nucleotide polymorphisms, or SNPs) that disrupt their coding region and destroy their function. Because these genes coexist with their intact counterparts in the human population, in contrast to hundreds of other pseudogenes shared by all human beings (and are thus called segregating pseudogenes, or SPGs), they are promising candidates for explaining human variance in odor detection. Individuals carry two alleles of a particular gene, one inherited from each parent. You could have two copies of the same allele (homozygous) or two different copies (heterozygous). The researchers reasoned that SPGs could generate millions of intact/disrupted combinations of alleles that a person can inherit at the dozens of SPG loci (their genotype), thus creating a huge diversity of olfactory sensitivities in the population. For example, a person with two active alleles might be hyperosmic to an odor, while someone with two nonfunctional pseudogenes might be anosmic.

To test this possibility, the researchers recruited 377 volunteers for a “phenotype–genotype” association study. Focusing on the candidate olfactory receptor SPGs, they asked whether there was a relationship between participants' sensitivity to four selected odorants (their phenotypes) and the various genotypes. As participants sniffed vials filled with isoamyl acetate (which smells like pear or banana), isovaleric acid (the classic sweaty odorant), carvone (spearmint), and cineole (eucalyptus), the researchers measured their detection thresholds (the lowest odor concentration an individual could accurately detect).

All the odors produced similar results in terms of the range of participants' sensitivities, with some individuals unable to detect the highest concentrations (anosmics), most people able to detect medium concentrations, and some able to detect the lowest (hyperosmics). Women in the study showed somewhat better sensitivity than the men, but age, smoking habits, and ethnic origin did not affect the results.

In the genetic association analysis, looking for correlations between olfactory thresholds and olfactory receptor genotypes, the researchers found a strong signal between sensitivity to isovaleric acid and one gene, *OR11H7P*. In keeping with established evidence that specific anosmia is a recessive trait (that is, requires two damaged alleles), the researchers found that participants carrying two copies of the disrupted *OR11H7P* allele (the pseudogene) were mostly numb to low quantities of isovaleric acid. In contrast, those with heightened sensitivity to the compound were more likely to have at least one intact allele.

Having found a variant to explain isovaleric acid sensitivity, the researchers wanted to make sure it could actually interact with this molecule. To do this, they used an assay designed to test the functional expression of olfactory receptors in frog oocytes. Using the intact allele, the pseudogenized allele, and three randomly chosen nondisrupted olfactory receptors, the researchers tested a range of isovaleric acid concentrations. Only the intact *OR11H7P* allele produced a response to isovaleric acid. (Two olfactory receptors that flank *OR11H7P* responded to similar concentrations, consistent with evidence that related olfactory receptors bind similar odorants.)

While this variant clearly plays a role in human olfactory variability, it doesn't explain all the variation observed in the study. The researchers also found a general olfactory effect, with individuals responding with similar acuity to all the odors. They conclude that hyperosmia is a complex trait that is likely modulated by a combination of genetic and environmental factors. It may be that genetic polymorphisms in individual receptors, combined with variations in fundamental olfactory pathways in the periphery and the brain, mediate the remarkable range of olfactory sensitivities in the human population. Thus, your ability to detect various odors might be enhanced if other aspects of your olfactory system are particularly efficient.

Although the molecular and cellular processes that guide olfactory perception—from odor detection to the representation of different odors in the brain—have recently received considerable scrutiny, crucial elements still remained obscure. With the link between a gene variant and differences in human olfactory sensitivity now demonstrated, researchers can further explore the genetic basis of the first step in this enigmatic sensory pathway.

